# Gene Therapy Potential for Genetic Disorders of Surfactant Dysfunction

**DOI:** 10.3389/fgeed.2021.785829

**Published:** 2022-01-14

**Authors:** Ashley L. Cooney, Jennifer A. Wambach, Patrick L. Sinn, Paul B. McCray

**Affiliations:** ^1^ Department of Pediatrics, The University of Iowa, Iowa City, IA, United States; ^2^ Pappajohn Biomedical Institute and the Center for Gene Therapy, The University of Iowa, Iowa City, IA, United States; ^3^ Edward Mallinckrodt Department of Pediatrics, Washington University School of Medicine, St. Louis, MO, United States

**Keywords:** surfactant deficiency, viral vectors, alveoli, pulmonary disease, AEC2, ATII, AT2

## Abstract

Pulmonary surfactant is critically important to prevent atelectasis by lowering the surface tension of the alveolar lining liquid. While respiratory distress syndrome (RDS) is common in premature infants, severe RDS in term and late preterm infants suggests an underlying genetic etiology. Pathogenic variants in the genes encoding key components of pulmonary surfactant including surfactant protein B (SP-B, *SFTPB* gene), surfactant protein C (SP-C, *SFTPC* gene), and the ATP-Binding Cassette transporter A3 (ABCA3, *ABCA3* gene) result in severe neonatal RDS or childhood interstitial lung disease (chILD). These proteins play essential roles in pulmonary surfactant biogenesis and are expressed in alveolar epithelial type II cells (AEC2), the progenitor cell of the alveolar epithelium. SP-B deficiency most commonly presents in the neonatal period with severe RDS and requires lung transplantation for survival. *SFTPC* mutations act in an autosomal dominant fashion and more commonly presents with chILD or idiopathic pulmonary fibrosis than neonatal RDS. ABCA3 deficiency often presents as neonatal RDS or chILD. Gene therapy is a promising option to treat monogenic lung diseases. Successes and challenges in developing gene therapies for genetic disorders of surfactant dysfunction include viral vector design and tropism for target cell types. In this review, we explore adeno-associated virus (AAV), lentiviral, and adenoviral (Ad)-based vectors as delivery vehicles. Both gene addition and gene editing strategies are compared to best design treatments for lung diseases resulting from pathogenic variants in the *SFTPB, SFTPC,* and *ABCA3* genes*.*

## Introduction

Pulmonary surfactant is a complex mixture of phospholipids and proteins that is secreted into the alveolar space, reduces surface tension, prevents end expiratory alveolar collapse, and is required for gas exchange. Surfactant is synthesized in lamellar bodies, specialized intracellular organelles derived from lysosomes in alveolar epithelial type II cells (AEC2, aka ATII or AT2 cells). Phospholipids are transported into the lamellar bodies by ABCA3 and assemble with surfactant proteins B and C to form surfactant. The lamellar bodies are released into the alveolar lumen via exocytosis. Pathogenic variants in genes that encode key components of pulmonary surfactant include surfactant protein B (SP-B, *SFTPB* gene), surfactant protein C (SP-C, *SFTPC* gene), and the ATP-binding cassette transporter A3 (ABCA3, *ABCA3* gene) and are leading inherited causes of neonatal respiratory distress syndrome (RDS) and childhood interstitial lung disease (chILD) (Garmany et al., 2008). Treatments for these monogenic pulmonary diseases are limited and non-specific, and for many patients, lung transplant may be the only option for survival beyond the first months of life (Wambach et al., 2014). Recent advances offer renewed opportunities to develop gene therapies for genetic disorders of surfactant dysfunction resulting from pathogenic variants in *SFTPB, SFTPC,* and *ABCA3.* There is no “one size fits all” approach for developing gene therapy vectors for SP-B, SP-C, and ABCA3 deficiencies. Non-viral vectors (i.e., plasmid DNA, nanoparticles, and or *in vitro* transcribed mRNA) and Epstein-Barr based plasmid DNA each have important qualities and have advanced the lung gene transfer field (reviewed in (Stribling et al., 1992; Tu et al., 2000)). This review will focus on adeno-associated virus (AAV), lentiviral, or adenoviral (Ad)-based delivery vehicles for a gene addition approach or delivery of gene editing tools to complement or repair disorders of surfactant dysfunction, as well as appropriate models to assess gene transfer efficacy. Our goal for this review is to introduce potential viral vector-mediated gene therapy options for surfactant diseases and provide enough details about each deficiency to highlight why gene therapy may be particularly challenging for this disease class.

### Surfactant Composition and Metabolism

**FIGURE 1:**
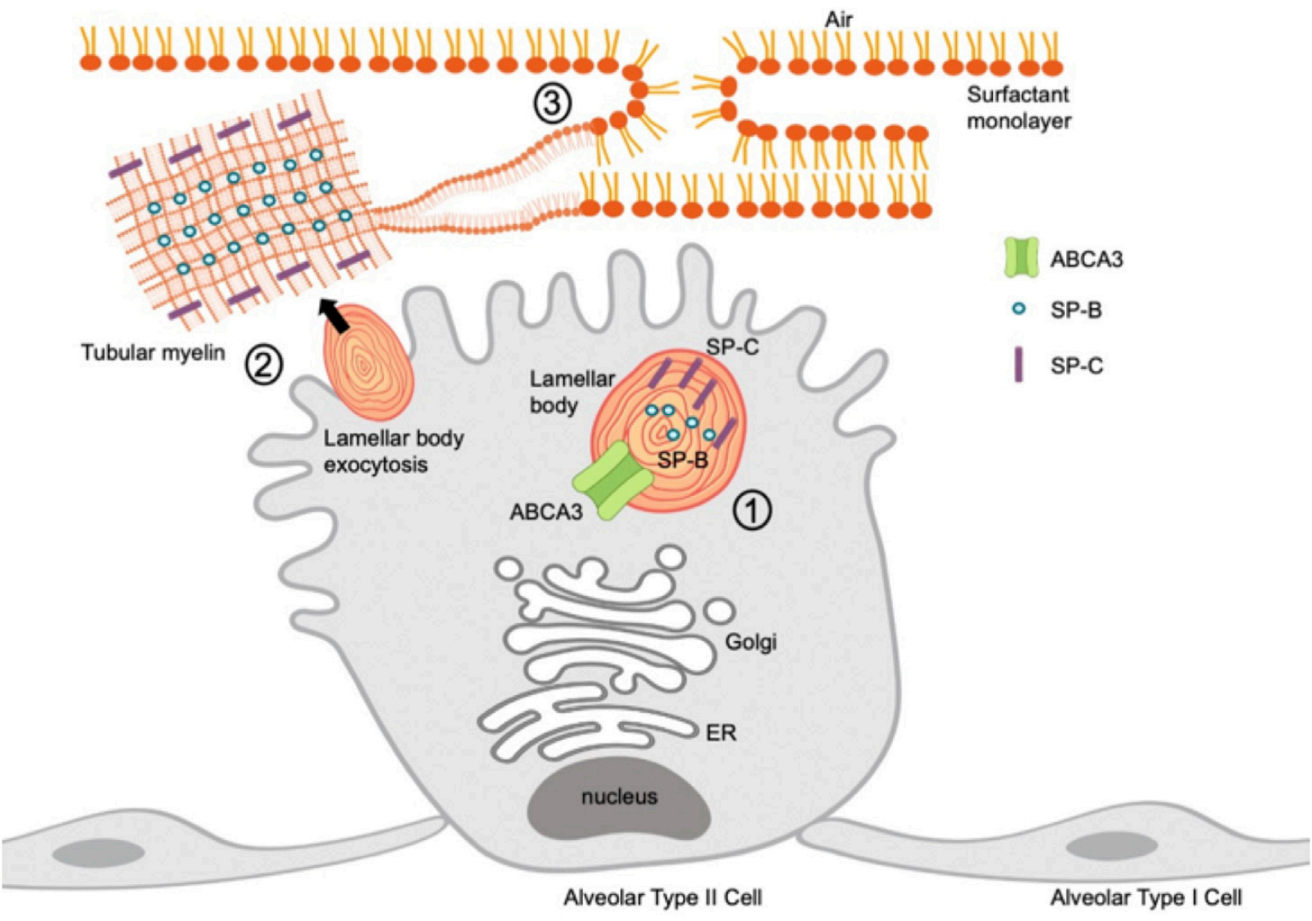
Schematic of SP-B, SP-C, and ABCA3 protein localization in an alveolar type II cell. 1) ABCA3 transports phospholipids into lamellar bodies; SP-B and SP-C provide support during surfactant assembly. 2) Lamellar bodies undergo exocytosis from alveolar type II cell and unravel into tubular myelin. 3) Tubular myelin disassembles into a surfactant monolayer through adsorption into a film at an air-liquid interface.

Pulmonary surfactant production is a highly regulated process of synthesis, secretion, degradation, and recycling. Surfactant is composed of 90% phospholipids, specifically phosphatidylcholine and phosphatidylglycerol, and cholesterol, and 10% surfactant proteins A, B, C, and D (Agassandian and Mallampalli, 2013). These components are synthesized in the endoplasmic reticulum in AEC2s and assembled and stored in lamellar bodies (Figure 1). Surfactant proteins B and C play a role in reducing surface tension while surfactant proteins A and D are collectins and play important roles in innate immunity (Pastva et al., 2007). Surfactant is released from lamellar bodies via exocytosis into the alveolar lumen. Upon release, the lamellar bodies unwind to form tubular myelin, an ordered structure which forms a film at the air-liquid interface (Cañadas et al., 2020).

SP-B, SP-C, and ABCA3 each play distinct roles in surfactant production and homeostasis. SP-B is a hydrophobic protein required for surfactant formation and is a key structural support, conferring surface tension lowering properties with phospholipid molecules to enhance surfactant spreading (Cochrane and Revak, 1991). SP-C enhances adsorption at the air-liquid interface to reduce surface tension. SP-C also plays an immunomodulatory role in clearing pulmonary infections, as SP-C deficient mice exhibit inflammation upon loss of SP-C (Glasser et al., 2009). ABCA3 is involved in lamellar body formation and phospholipid transport. Loss of ABCA3 results in surfactant lacking phosphatidylcholine and increased surface tension (Garmany et al., 2006).

## SP-B Deficiency

Newborns with SP-B deficiency typically present with RDS shortly after birth and die of progressive respiratory failure in the first few months of life without a lung transplant. A few children with biallelic *SFTPB* variants and chronic respiratory insufficiency have been reported (Dunbar et al., 2000). SP-B deficiency is an autosomal recessive disease and the most common pathogenic variant p. Pro133Glnfs*95 (previously known as ‘121ins2’) results in a frameshift and nonsense-mediated decay of the mRNA transcript. Treatment with exogenous surfactant enriched in SP-B protein is ineffective (Thompson, 2001) and lung transplant remains infrequent due to inavailability of suitable neonatal donor lungs (Eldridge et al., 2017). Gene addition (Barnett et al., 2017; Leibel et al., 2019; Kang et al., 2020) and gene editing approaches (Mahiny et al., 2015; Jacob et al., 2017) have demonstrated that complementing SP-B expression in AEC2s restores the phenotypic defect *in vitro* and *in vivo*. Although gene editing approaches may be successful, the diversity of pathogenic variants suggests that a gene addition approach may be a more near term goal for SP-B deficiency.

**FIGURE 2:**
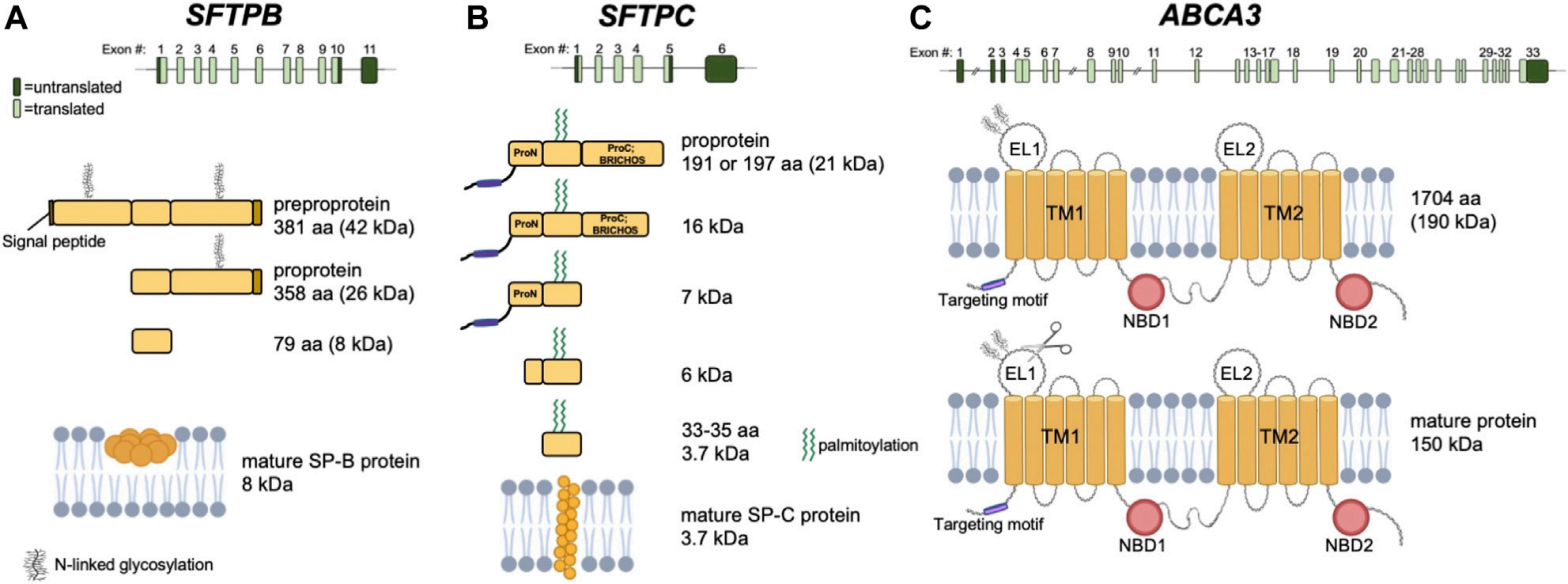
**(A)** Top panel: Exon representation of *SFTPB* on chromosome 2. Middle panels: progression of protein processing. Glycosylation at residues 129-131 and 311-313 occurs in ER. Bottom panel: yellow circles indicate oligomer of at least 6 homo-dimers inserted into a lipid bilayer. **(B)** Top panel: Exon representation of *SFTPC* on chromosome 8. Middle panels: progression of protein processing. Palmitoylation occurs in the Golgi apparatus. Cleavage events resulting in shortened protein forms are the result of Cathepsin H and Pepsinogen C enzymatic activities. Bottom panel: yellow circles indicate mature membrane bound protein. **(C)** Top panel: Exon representation of *ABCA3* on chromosome 16. Middle panel: N-linked glycosylation at positions N124 and N140 indicated by N-linked glycosylation symbol. Bottom panel: N-terminus is proteolytically cleaved by cathepsins L and B, as indicated by scissors icon in EL1. TM, transmembrane domain; EL, extracellular loop; NBD, nucleotide binding domain; aa, amino acid.

SP-B plays a major role in the assembly and function of pulmonary surfactant and is required for proper lamellar body biogenesis in AEC2s (Figure 1). It commonly exists as a homodimer and is a small, 79 amino acid hydrophobic protein that permeabilizes, cross-links, mixes, and fuses cell membranes. Mature SP-B results from proteolytic modifications involving the 381 amino acid preproSP-B peptide followed by additional glycosylation and proteolytic events of proSP-B (Guttentag, 2008) (Figure 2A). Pathogenic *SFTPB* variants that disrupt glycosylation or proteolytic cleavage sites have been identified (Lin et al., 2000). While both viral and nonviral delivery strategies offer promise, here we will focus on viral vectors.

### Gene Therapy Approach: Adeno-Associated Virus Vectors

AAV vectors have gained interest with increasing clinical applications following the FDA approval of Luxturna (Maguire et al., 2008), Zolgensma (Mendell et al., 2017), and promising studies for Duchenne muscular dystrophy (Duan, 2018). AAV vectors are episomal, have excellent safety profiles, and persist long-term in mitotically quiescent cells. Additionally, AAV can be produced to high titers and has a scalability to produce clinical grade vector (Selvaraj et al., 2021). However, the ∼4.7 kb packaging capacity limit is challenging for large transgenes. The relatively small coding sequence of SP-B (∼1.2 kb) makes AAV a feasible vector candidate. Indeed, Kang et al. used AAV to deliver *SFTPB* and restored surfactant production and improved survival in the conditional lethal SP-B knockout mouse model (Kang et al., 2020). AAV6 carrying a *proSFTPB* cDNA was delivered to conditional SP-B null mice, which typically die within 2 days of birth due to respiratory failure. In this study, survival increased to ∼200 days. Additionally, lamellar body formation was restored and lung structure and function were improved. Other approaches such as delivering truncated versions of SP-B demonstrated increased survival in SP-B deficient mice. These studies also revealed that the C-terminal portion of the protein is important for surfactant formation (Akinbi et al., 1997).

When designing a gene therapy vector, an important goal is the efficient delivery to and the persistent expression of the transgene in a target cell type. Endpoints to be quantified include the transduction efficiency, transgene expression (mRNA and protein), and functional correction. To achieve this with AAV, capsid selection is necessary for transducing AEC2s. AAV2, AAV6, and AAV6 variant capsids transduce AEC2 organoid models (Meyer-Berg et al., 2020) as well as both airway and alveolar epithelial cells (Kang et al., 2020). The route of administration is an important consideration for successfully transducing target cells of interest. AAV9 administered intravenously transduces heart, skeletal muscle, and pancreas with better efficacy compared to an AAV8 capsid (Inagaki et al., 2006). Whether topical lung delivery or systemic delivery is superior for AEC2 transduction remains to be determined.

Following efficient transduction of AEC2s, achieving persistent expression in the lung likely will require integration in a progenitor cell population. AAV is generally considered to be non-integrating, but its persistence in AEC2s is unknown. Incorporating an integrating system such as the piggyBac transposon into AAV is a potential strategy to achieve persistent expression in the alveoli (Cooney et al., 2015; Brommel et al., 2020). AEC2s are an important progenitor cell in the alveolus (Olajuyin et al., 2019) and while they retain secretory functions for surfactant production, they are also critical for alveolar homeostasis, through self-renewal or differentiation to alveolar type I cells (Mason and Williams, 1977; Fehrenbach, 2001). Lastly, promoter and polyadenylation (pA) tail choice is important to consider. Promoter size is another key factor in decision making, specifically a short promoter with sufficient activity. Synthetic promoters such as F5Tg83 and a short polyA have been created to maximize space within AAV vectors (Yan et al., 2015). AEC2-specific promoters for cell specific expression of SP-B and ABCA3 have yet to be evaluated. The SP-C promoter is a candidate for directing AEC2 specific gene expression (Degiulio et al., 2010).

### Gene Therapy Approach: Lentiviral Vectors

Lentiviral vectors demonstrated therapeutic efficacy in *ex vivo* gene transfer to hematopoietic stem cells for as ADA-SCID (Kohn et al., 2021), β-Thalassemia (Thompson et al., 2018), and Wiskott-Aldrich syndrome (Sereni et al., 2019). Insertional mutagenesis is a potential risk with any integrating vector. The functional consequences of lentiviral vector integration has received considerable attention (reviewed in (Schlimgen et al., 2016; Milone and O’Doherty, 2018)). However, using the current generation of self-inactivating vectors, the clonal expansion of corrected cells has not been observed following *ex vivo* or systemic delivery of lentiviral vectors. The risk versus benefit must be taken into consideration when evaluating the use of an integrating vector. Lentiviral vectors are recognized for their ability to transduce both dividing and non-dividing cells, integrate for long-term expression, and allow readministration without blocking immune responses (Sinn et al., 2008). Lentiviral vectors encoding *SFTPB* delivered to alveolar organoids conferred SP-B expression and reversed the surfactant deficiency phenotype *in vitro* (Leibel et al., 2019; Munis et al., 2021). A key consideration in designing a therapeutic lentiviral vector for SP-B deficiency is envelope selection to transduce the appropriate cell type. Vesicular stomatitis virus glycoprotein (VSV-G) is the most common envelope used to pseudotype retroviruses and is reported to transduce AEC2s (Borok et al., 2001). Other envelopes such as baculovirus GP64 (Sinn et al., 2012) and Sendai virus F/HN (Griesenbach et al., 2012) also transduce alveolar epithelia. Options for promoter choice include cell type specific or constitutive elements. Production of clinical grade vector for *in vivo* somatic cell targeting represents a challenge, but advances in lentivirus manufacturing (Valkama et al., 2018; Martínez-Molina et al., 2020; Soldi et al., 2020) make this a promising therapeutic for SP-B deficiency.

## SP-C Associated Interstitial Lung Disease

SP-C is a 35-amino acid hydrophobic protein that organizes with SP-B to lower the surface tension of surfactant (Figure 1). The *SFTPC* locus spans ∼3,500 bp and is expressed from six exons on chromosome 8. A 197-amino acid protein is first produced with an N-terminal propeptide domain and C-terminal BRICHOS domain (pro-SPC). As the mature protein is processed, the N-terminal domain is cleaved from the proSPC form to SP-C. The mature protein has a shortened N-terminal domain, linker, and C-terminal BRICHOS domain (Figure 2B).

Pathogenic variants in *SFTPC* can arise *de novo* (∼50% of cases) or are inherited (∼50% of cases) (Nogee et al., 2002). A pathogenic splicing variant in *SFTPC* within the first base of intron 4 (c.460+1G > A) was first reported in 2001 in an infant and mother with interstitial pneumonitis (Nogee et al., 2001). Subsequently, additional pathogenic *SFTPC* variants have been identified, the most frequent of which is p. I73T (Cameron et al., 2005). Individuals with pathogenic *SFTPC* variants most commonly present with sporadic or familial interstitial lung disease as infants, children, or adults, and less frequently with neonatal RDS (Nogee et al., 2002). The disease course is highly variable with some infants presenting with severe respiratory failure requiring lung transplant, others exhibiting chronic disease managed with long-term mechanical ventilation (Liptzin et al., 2015), and others remaining relatively asymptomatic (Thomas et al., 2002). Genotype alone is not predictive of disease presentation, severity, and or course. Pathogenic *SFTPC* variants located in the C-terminal BRICHOS domain, which acts as a chaperone to promote protein stability, result in increased endoplasmic reticulum stress, inflammation, and spontaneous pulmonary fibrosis in a murine model (Katzen et al., 2019). Non-BRICHOS variants, which include p. I73T, result in defective AEC2 macroautophagy, inflammation, remodeling, and fibrosis (Nureki et al., 2018).

SP-C associated interstitial lung disease is a gain-of-toxic-function disease inherited in an autosomal dominant pattern with variable penetrance (Thomas et al., 2002). Expression of a mutant *SFTPC* allele is responsible for the surfactant dysfunction phenotype. The resultant gain-of-toxic-function requires silencing of the mutant allele. Therefore, a gene addition approach is not an option. An allele specific gene knockout or knockdown approach can be used to disrupt and inactivate or reduce the abundance of the mutant *SFTPC* product. CRISPR/Cas9 nuclease-mediated gene knockout has been validated in mice *in utero* by targeting the p.173T allele, resulting in improved lung morphology, and increased survival of offspring (Alapati et al., 2019). RNAi and antisense RNA strategies can also be envisioned.

### Gene Editing Approach: AAV Vectors

Common gene editing approaches, such as CRISPR/Cas9, may be employed using either homologous recombination or non-homologous end joining approaches to repair or inactivate the mutant allele. Alternatively, the advent of cytosine and adenine base editors and prime editing may allow single base changes without causing indels (Levy et al., 2020). AAV is commonly used to deliver gene editing tools, including incorporating a split-intein system to allow for co-delivery of cassettes larger than the AAV packaging capacity. Alternatively, the smaller *Staphylococcus aureus* Cas9 can be delivered with a sgRNA using a single AAV vector (Lau and Suh, 2017). As with developing a gene editing treatment for any disease, an individualized approach may be required for the array of pathogenic *SFTPC* variants that cause interstitial lung disease.

## ABCA3 Deficiency

ABCA3 deficiency, an autosomal recessive disorder, and is caused by pathogenic variants in *ABCA3*. Biallelic variants in *ABCA3* cause severe neonatal RDS, chILD, and adult pulmonary fibrosis (Shulenin et al., 2004; Klay et al., 2020; Tomer et al., 2021). Infants with biallelic *ABCA3* frameshift or nonsense variants present with neonatal RDS at birth and die within the first year of life without lung transplant (Wambach et al., 2014). The respiratory phenotypes and disease courses for individuals with *ABCA3* missense variants are variable and include neonatal RDS and interstitial lung disease.

ABCA3 is a phospholipid transporter located at the lysosomal-derived lamellar body limiting membrane and plays a critical role in surfactant assembly and lamellar body formation (Figure 1). The *ABCA3* cDNA encodes a 1,704 amino acid polypeptide. Mature ABCA3 protein is folded in the endoplasmic reticulum and undergoes glycosylation in the Golgi (Beers and Mulugeta, 2017). A second post-translational modification involves the N-terminal proteolytic cleavage of the 190 kD protein, shortening the protein to 150 kD (Engelbrecht et al., 2010) (Figure 2C). The lamellar bodies from infants and children with ABCA3 deficiency are small with dense bodies and are described as having a “fried-egg appearance” (Wert et al., 2009). Two mechanistic classes of *ABCA3* missense variants have been identified: disruption of intracellular trafficking and impaired phospholipid transport (Matsumura et al., 2006; Denman et al., 2018; Wambach et al., 2020). The most common pathogenic variant is p. E292V, a missense variant that impairs phospholipid transport (Wambach et al., 2016). This variant is commonly associated with chILD (Wambach et al., 2014) and is present in 0.4% of individuals in Genome Aggregation database (gnomad.broadinstitute.org, accessed September 2021).

The overall goal for gene therapy to treat loss-of-function diseases is to efficiently and persistently (long-term) express ABCA3 at physiological levels in AEC2s. A gene addition strategy involves complementing the loss of function with a full length *ABCA3* cDNA. Because most *ABCA3* variants are rare and private, this poses a challenge for developing variant-specific gene editing or targeted drug approaches. The size constraints of some viral vectors present challenges for a transgene as large as *ABCA3*.

### Gene Therapy Approach: Lentiviral Vectors

Gene addition of *ABCA3* cDNA using lentiviral vectors is a promising therapeutic approach. Lentiviral vectors have a packaging capacity of at least 7.5 kb and could readily accommodate the 5.1 kb *ABCA3* transgene and promoter. Additionally, lentiviral vectors are amenable to pseudotyping with various envelopes to modify tropism. As discussed in the lentiviral section of SP-B deficiency, envelope, promoter, and polyadenylation signal are important considerations in designing a gene therapy vector for ABCA3 deficiency. The integrating properties of lentiviral vectors make this vector class an attractive option for ABCA3 deficiency.

### Gene Therapy Approach: Adeno-Associated Virus ectors

Given that the *ABCA3* 5.1 kb cDNA size surpasses the 4.7 kb packaging limit of AAV vectors, an AAV approach to deliver the full length *ABCA3* is challenging with current technologies. Approaches using AAV to restore ABCA3 deficiency can be modeled from other diseases, including insertion of a partial super exon cDNA as described for another ABC transporter protein, and cystic fibrosis transmembrane conductance regulator (*CFTR* also know as *ABCC7*) (Bednarski et al., 2016). A dual vector approach using split inteins has been employed to deliver gene editing tools, specifically splitting the Cas9 transgene between two AAV vectors which are joined by a C-terminal and N-terminal inteins (Tornabene et al., 2019); however, this approach may not be feasible in proteins with multiple transmembrane domains. Further studies are required to investigate whether *ABCA3* could be delivered using such dual vector platforms.

### Gene Therapy Approach: Adenoviral Vectors

Adenoviral (Ad)-based vectors provide robust expression and a relatively large carrying capacity (∼10 kb). Additional features of Ad vectors are: 1) they transduce both dividing and non-dividing cells, 2) they have broad tissue tropism, and 3) they are scalable for clinical platforms. Still, their greatest hurdle is the innate and adaptive immune responses which can clear transduced cells. In efforts to overcome limitations due to immune responses, helper-dependent Ad (HDAd) vectors were created by deleting all viral genes, leaving only a packaging signal. The required viral components are provided by a helper virus *in trans* during virus production. A second limitation is that Ad is not an integrating vector. However, Ad-based delivery of gene editing tools has shown long-term phenotypic correction of Hemophilia B in a mouse model (Stephens et al., 2019). Furthermore, numerous studies have incorporated transposon systems such as Sleeping Beauty and piggyBac to create hybrid integrating adenoviral vectors (Cooney et al., 2015; Boehme et al., 2016). Ad-based vectors remain a candidate platform for gene therapy and vaccine developments. An advantage for the production of Ad-based vectors is their ability to be grown to high titers and stringently purified (Danthinne and Imperiale, 2000; Nadeau and Kamen, 2003).

Given the 5.1 kb *ABCA3* transgene, Ad-based vectors have been extensively used to study ABCA3 complementation and the impact of various variants. For example, Ad vectors carrying *ABCA3* variants including p. L101P (mistrafficking mutant) and p. E292V (impaired phospholipid transport mutant) were used to functionally characterize mutant effects in a pulmonary epithelial cell line (A549 cells) (Wambach et al., 2016; Hu et al., 2020; Wambach et al., 2020). These studies helped establish how the proteins from each mutant mechanistic class traffic through the cell, affect lamellar body formation, and transport phosphatidylcholine. Identification of variant-specific mechansims may inform disease course or therapeutic approach.

## Preclinical Model Systems to Validate Gene Therapy Strategies for Surfactant Deficiencies

Immortalized pulmonary epithelial cell lines with characteristics of AEC2s such as A549 cells are a first line model system because they are easy to passage and maintain, form lamellar body-like structures which can be visualized by electron microscopy, and are readily transduced by viral vectors. A549 *ABCA3* ^−/−^ cells are established and lack well-developed lamellar body-like structures (Wambach et al., 2020). However, a major limitation for tumor-derived cell lines such as A549 is that they do not produce surfactant. Alveolar cells need to differentiate in order to produce surfactant and express SP-C, a common marker used to identify AEC2s.

Alveolar epithelial cell organoids derived from human embryonic or pluripotent stem cells generate alveolospheres in culture which display functional properties of AEC2s. These cells can proliferate, differentiate, and be modified to encompass surfactant dysfunction phenotypes (Jacob et al., 2017; Jacob et al., 2019). iPSC organoids derived from a patient with SP-B deficiency mirrored the disease phenotype including decreased surfactant production. This defect was restored when transduced with a lentiviral vector carrying *SFTPB*, including proper lamellar body formation (Leibel et al., 2019). In total, AEC2 organoids are a useful model to assess strategies to restore function of surfactant components.

Measuring restoration of surfactant function is challenging. Surfactant proteins B and C are typically quantified to confirm the presence of surfactant proteins in cell lysates or secretions. The measurement of phospholipid transport using dipalmitylphosphatidylcholine (DPPC) release is one method to assess surfactant production (Jacob et al., 2017). Instruments such as the pulsating bubble surfactometer can be used to measure the surface tension lowering properties of cell secretions.

Mouse models of each of these surfactant-based genetic diseases have been generated. SP-B knockout mice develop severe respiratory distress and die within hours of birth and exhibit dense, abnormal lamellar body-like organelles (Clark et al., 1995; Stahlman et al., 2000). Restoring SP-B using an AAV vector improved survival in conditional SP-B knockout mice (Kang et al., 2020). Alternatively, SP-C knockout mice are viable and can survive to adulthood. They produce lamellar bodies and surfactant proteins A, B, and D. Phenotypic responses include decreased stability of surfactant at low volumes, pneumonitis and emphysema (Glasser et al., 2001; Whitsett and Weaver, 2002). *ABCA3* knockout mice have a similar outcome as SP-B knockout mice and do not survive (Ban et al., 2007; Fitzgerald et al., 2007). Conditionally null *ABCA3* mice have also been generated (Besnard et al., 2010).

## Concluding Remarks

Gene based therapies for disorders of surfactant dysfunction resulting from pathogenic variants in *SFTPB, SFTPC,* and *ABCA3* are now realizable. Generating a delivery vector for either a gene addition or gene editing approach requires the careful consideration of vector design, transgene expression cassette, preclinical models used, and assays to assess the correction of surfactant production and function.
